# Chemically induced reprogramming to reverse cellular aging

**DOI:** 10.18632/aging.204896

**Published:** 2023-07-12

**Authors:** Jae-Hyun Yang, Christopher A. Petty, Thomas Dixon-McDougall, Maria Vina Lopez, Alexander Tyshkovskiy, Sun Maybury-Lewis, Xiao Tian, Nabilah Ibrahim, Zhili Chen, Patrick T. Griffin, Matthew Arnold, Jien Li, Oswaldo A. Martinez, Alexander Behn, Ryan Rogers-Hammond, Suzanne Angeli, Vadim N. Gladyshev, David A. Sinclair

**Affiliations:** 1 Paul F. Glenn Center for Biology of Aging Research, Department of Genetics, Blavatnik Institute, Harvard Medical School (HMS), Boston, MA 02115, USA; 2 Division of Genetics, Department of Medicine, Brigham and Women’s Hospital, Harvard Medical School, Boston, MA 02115, USA; 3 Belozersky Institute of Physico-Chemical Biology, Moscow State University, Moscow 119234, Russia; 4 Molecular and Biomedical Sciences, University of Maine, Orono, ME 04467, USA; 5 Department of Biology and Chemistry, Massachusetts Institute of Technology, Cambridge, MA 02142, USA

**Keywords:** reprogramming, rejuvenation medicine, information theory of aging, small molecules, epigenetics

## Abstract

A hallmark of eukaryotic aging is a loss of epigenetic information, a process that can be reversed. We have previously shown that the ectopic induction of the Yamanaka factors OCT4, SOX2, and KLF4 (OSK) in mammals can restore youthful DNA methylation patterns, transcript profiles, and tissue function, without erasing cellular identity, a process that requires active DNA demethylation. To screen for molecules that reverse cellular aging and rejuvenate human cells without altering the genome, we developed high-throughput cell-based assays that distinguish young from old and senescent cells, including transcription-based aging clocks and a real-time nucleocytoplasmic compartmentalization (NCC) assay. We identify six chemical cocktails, which, in less than a week and without compromising cellular identity, restore a youthful genome-wide transcript profile and reverse transcriptomic age. Thus, rejuvenation by age reversal can be achieved, not only by genetic, but also chemical means.

## INTRODUCTION

All life depends on the storage and preservation of information. In eukaryotes, there are two main repositories of information: the genome and the epigenome. Though these information repositories work interdependently to coordinate the production and operation of life’s molecular machinery, they are different in fundamental ways. Genetic information is digital and largely consistent across all cells in the body throughout an individual’s lifespan. In contrast, epigenetic information is encoded by a less stable digital-analog system, varying between cells and changing in response to the environment and over time.

At least a dozen “hallmarks of aging” are known to contribute to the deterioration and dysfunction of cells as they age [1, 2]. We and other researchers have gathered compelling evidence, from yeast to mammals, supporting the idea that a loss of epigenetic information, resulting in changes in gene expression, leads to the loss of cellular identity [3–7]. These findings are consistent with the Information Theory of Aging, which proposes that a decline in information, specifically epigenetic information, triggers a cascade of events, including mitochondrial dysfunction, inflammation, and cellular senescence [5, 7–9], leading to a progressive decline in cell and tissue function, manifesting as aging and age-related diseases. We have previously shown in mice that cell injuries, such as DNA double-strand breaks and cell crushing, promote epigenetic information loss, which can lead to what appears to be an acceleration of aging and age-related disease [7, 9].

Cellular senescence is a state of permanent cell cycle arrest that facilitates wound repair, tissue remodeling, and avoidance of cancer by halting proliferation in aged and damaged cells [10, 11]. Senescence is associated with alterations in cell morphology, chromatin architecture, and the release of inflammatory factors in a process referred to as the senescence-associated secretory phenotype (SASP). The transition to cellular senescence can be initiated by a loss of epigenetic information, as well as telomere shortening, irreparable DNA damage, and cytoplasmic DNA [7, 10-12]. The accumulation of senescent cells with age promotes inflammation and generates additional reactive oxygen species (ROS), both locally and across the organism, contributing to a broad range of age-related diseases, from macular degeneration, to increased blood pressure, to metabolic dysregulation [13].

Starting in 1962, Gurdon and others demonstrated that nuclei contain the necessary information to generate new individuals with normal lifespans [14–16]. In 2006, Takahashi and Yamanaka demonstrated that the expression of four transcription factors, OCT4, SOX2, KLF4, and c-MYC (collectively known as “OSKM”), reprograms the developmental potential of adult cells, enabling them to be converted into various cell types [17, 18]. These findings initiated the field of cell reprogramming, with a string of publications in the 2000s showing that the identity of many different types of adult cells from different species could be erased to become induced pluripotent stem cells, commonly known as “iPSCs” [17, 19–21].

The ability of the Yamanaka factors to erase cellular identity raised a key question: is it possible to reverse cellular aging *in vivo* without causing uncontrolled cell growth and tumorigenesis? Initially, it didn’t seem so, as mice died within two days of expressing OSKM. But work by the Belmonte lab, our lab, and others have confirmed that it is possible to safely improve the function of tissues *in vivo* by pulsing OSKM expression [22, 23] or by continuously expressing only OSK, leaving out the oncogene c-MYC [7, 8]. In the optic nerve, for example, expression of a three Yamanaka factor combination safely resets DNA methylomes and gene expression patterns, improving vision in old and glaucomatous mice via a largely obscure mechanism that requires TET DNA demethylases [8]. Numerous tissues, including brain tissue, kidney, and muscle, have now been reprogrammed without causing cancer [7, 8, 22, 24, 25]. In fact, expression of OSK throughout the entire body of mice extends their lifespan [26]. Together, these results are consistent with the existence of a “back-up copy” of a youthful epigenome, one that can be reset via partial reprogramming to regain tissue function, without erasing cellular identity or causing tumorigenesis [7–9].

Currently, translational applications that aim to reverse aging, treat injuries, and cure age-related diseases, rely on the delivery of genetic material to target tissues. This is achieved through methods like adeno-associated viral (AAV) delivery of DNA and lipid nanoparticle-mediated delivery of RNA [7, 8, 27]. These approaches face potential barriers to them being used widely, including high costs and safety concerns associated with the introduction of genetic material into the body. Developing a chemical alternative to mimic OSK’s rejuvenating effects could lower costs and shorten timelines in regenerative medicine development [26, 28–31]. This advancement might enable the treatment of various medical conditions and potentially even facilitate whole-body rejuvenation [32, 33].

In this study, we developed and utilized novel screening methods including a quantitative nucleocytoplasmic compartmentalization assay (NCC) that can readily distinguish between young, old, and senescent cells [34, 35]. We identify a variety of novel chemical cocktails capable of rejuvenating cells and reversing transcriptomic age to a similar extent as OSK overexpression. Thus, it is possible to reverse aspects of aging without erasing cell identity using chemical rather than genetic means.

## RESULTS

### Nucleocytoplasmic compartmentalization (NCC) is disrupted in fibroblasts from old individuals and senescent cells

To identify small molecules that ostensibly reverse the effects of aging and senescence, we developed an efficient high-throughput system. Rather than relying on a limited set of genes that exhibit age-related changes and to ensure reliability and applicability across various cell types, we sought to develop an age-dependent assay that acted as a surrogate for cellular health and youthful gene expression patterns. To increase scalability and ease of use, we sought a fluorescence-based system that could be quantified in millions of cells per experiment via automated microscopy.

One of the most well-conserved physiological hallmarks of aging is deterioration of nucleocytoplasmic compartmentalization (NCC), which can be visualized as the leakage of nuclear proteins into the cytoplasm and failure of proteins to be imported into the nucleus [34, 35]. In neurons and astrocytes directly converted from fibroblasts of old humans, as well as old nematodes and rat brain tissue, the nuclear pore complex is deteriorated, leading to increased nuclear permeability and cytosolic protein aggregation [34–36].

**Figure 1:**
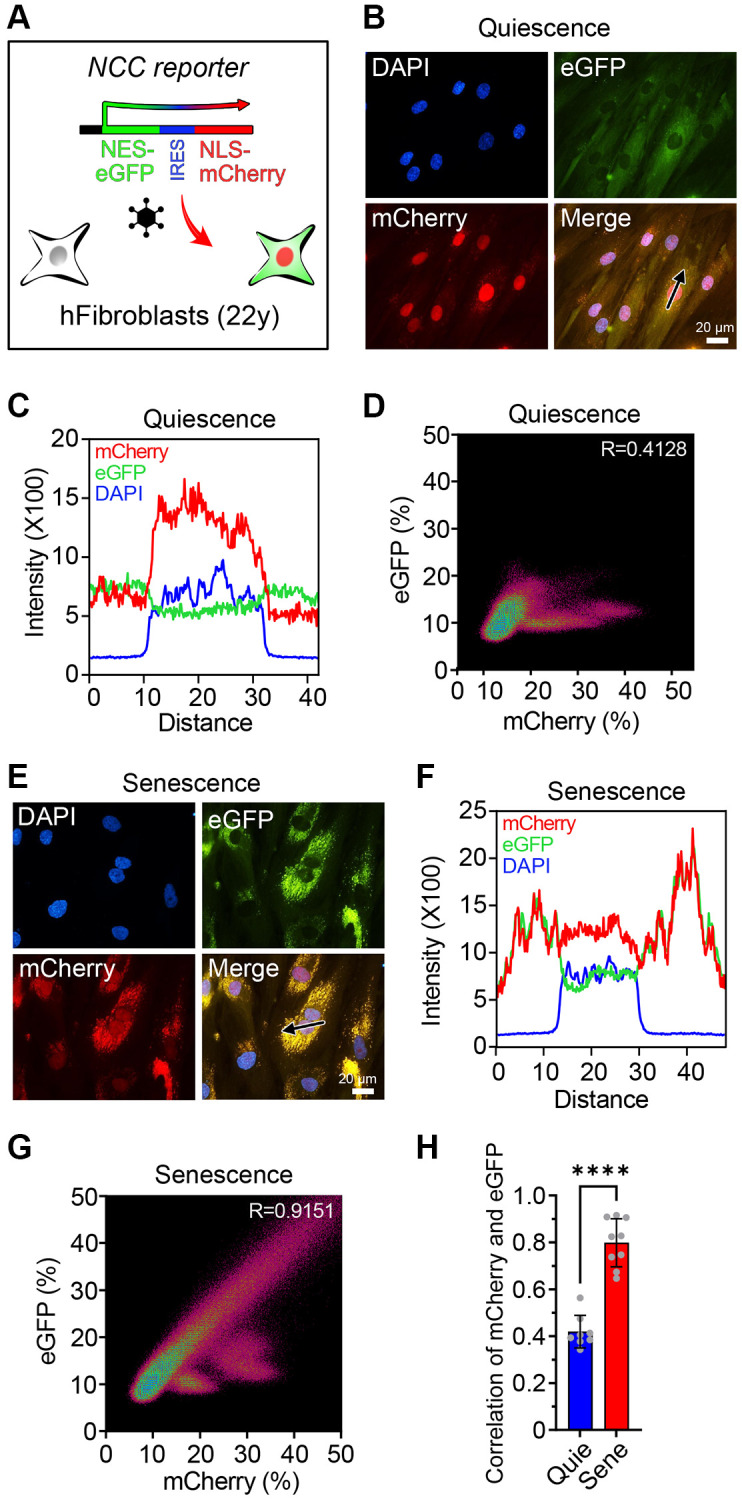
**The NCC reporter system to monitor cellular senescence.** (**A**) The NCC reporter system integrated in human fibroblasts. (**B**) NCC signals in quiescent fibroblasts. (**C**) Fluorescence intensity profiles corresponding to the path of the arrow in (**B**). (**D**) The colocalization of mCherry and eGFP signals in quiescent fibroblasts by Pearson correlation. (**E**) NCC signals in senescent fibroblasts. (**F**) Fluorescence intensity profiles corresponding to the path of the arrow in (**E**). (**G**) The colocalization of mCherry and eGFP signals in senescent fibroblasts by Pearson correlation. (**H**) Pearson correlation of quiescent and senescent fibroblasts. Data are mean ± SD. ^****^p < 0.0001. Two-tailed Student’s *t* test.

To monitor age-associated alterations in nuclear permeability, we introduced the NCC reporter system into human fibroblasts from a 22-year-old donor (Figure 1A). mCherry and eGFP were linked to nuclear localization signal (NLS) and nuclear export signal (NES), respectively. In healthy young fibroblasts, the cellular localization of these proteins distinctly separated, whereas in fibroblasts from either a 94-year-old donor or from a 14-year-old Hutchinson-Gilford progeria syndrome (HGPS) patient, the number and intensities of cytoplasmic mCherry puncta were higher than in fibroblasts from the normal 22-year-old donor (Supplementary Figure 1). Despite the difference, Z-factor analysis indicated that the system was not sufficiently robust for large-scale screening purposes, leading us to seek an alternative [37].

Cellular senescence is accompanied by substantial reorganization of the nuclear envelope and a breakdown in nucleocytoplasmic trafficking, including altered expression and degradation of Lamin B1, and the formation of cytoplasmic chromatin fragments (CCFs) [38–45]. Thus, we reasoned that senescent fibroblasts might produce a strong signal in the NCC reporter system, one that could be used for the screening of molecules to reverse epigenetic aging. Senescence can be induced in a variety of ways, including telomere erosion, oncogene expression, and DNA damage [13, 46]. Because replicative senescence advances the DNA methylation clock but DNA damage-induced senescence does not [46, 47], we reasoned that replicatively senescent cells might be more robust and reliable to find epigenetic age reversal cocktails than other types of senescent cells.

To avoid unintended false-rejuvenation effects caused by the expansion of a small percentage of replication-capable cells in the senescent population, all experiments were performed in low serum conditions that completely suppressed cell division [48]. In non, senescent, quiescent control fibroblasts, the mCherry and eGFP signals were clearly distinguishable (Figure 1B–1D). Senescent fibroblasts were generated by passaging ~40 times, each time with a 1:3–1:5 dilution with fresh media, until there was a complete absence of growth for two weeks, morphology changes characteristic of senescent cells, a dramatic increase in transcripts from the cell-cycle regulator p21 (CDKN1A), and other senescence-associated changes in gene expression (Supplementary Figure 2B, 2C). In the senescent fibroblasts, mCherry was aggregated in the cytoplasm and colocalized with eGFP (Figure 1E–1G), consistent with a previous report [34, 35]. Colocalization of the signals, as measured by Pearson correlation, was significantly higher in replicatively senescent cells compared to quiescent cells (Figure 1H). These experiments indicated that the NCC system could discern non-senescent from replicatively senescent cells, essentially in real time.

### Reversal of characteristics of cellular senescence by epigenetic reprogramming

To assess the applicability of the NCC system for detecting interventions that restore youthful functions and gene expression patterns, we first tested whether it could detect the effects of genetically mediated epigenetic age reversal. Ectopic expression of Yamanaka factors OCT4, SOX2, and KLF4 (OSK) restores youthful gene expression patterns, epigenetic age, and youthful functions to old cells and tissues [7, 8]. Our previously published reverse tetracycline-controlled transactivator (rtTA) module and the polycistronic OSK cistron under the control of a tetracycline-inducible promoter (Tet-on OSK) were transduced using lentivirus to create stable cell lines from the human fibroblasts and passaged until they reached replicative senescence. Treatment with doxycycline was sufficient to activate the OSK cassette in these fibroblasts (Supplementary Figure 2A).

**Figure 2:**
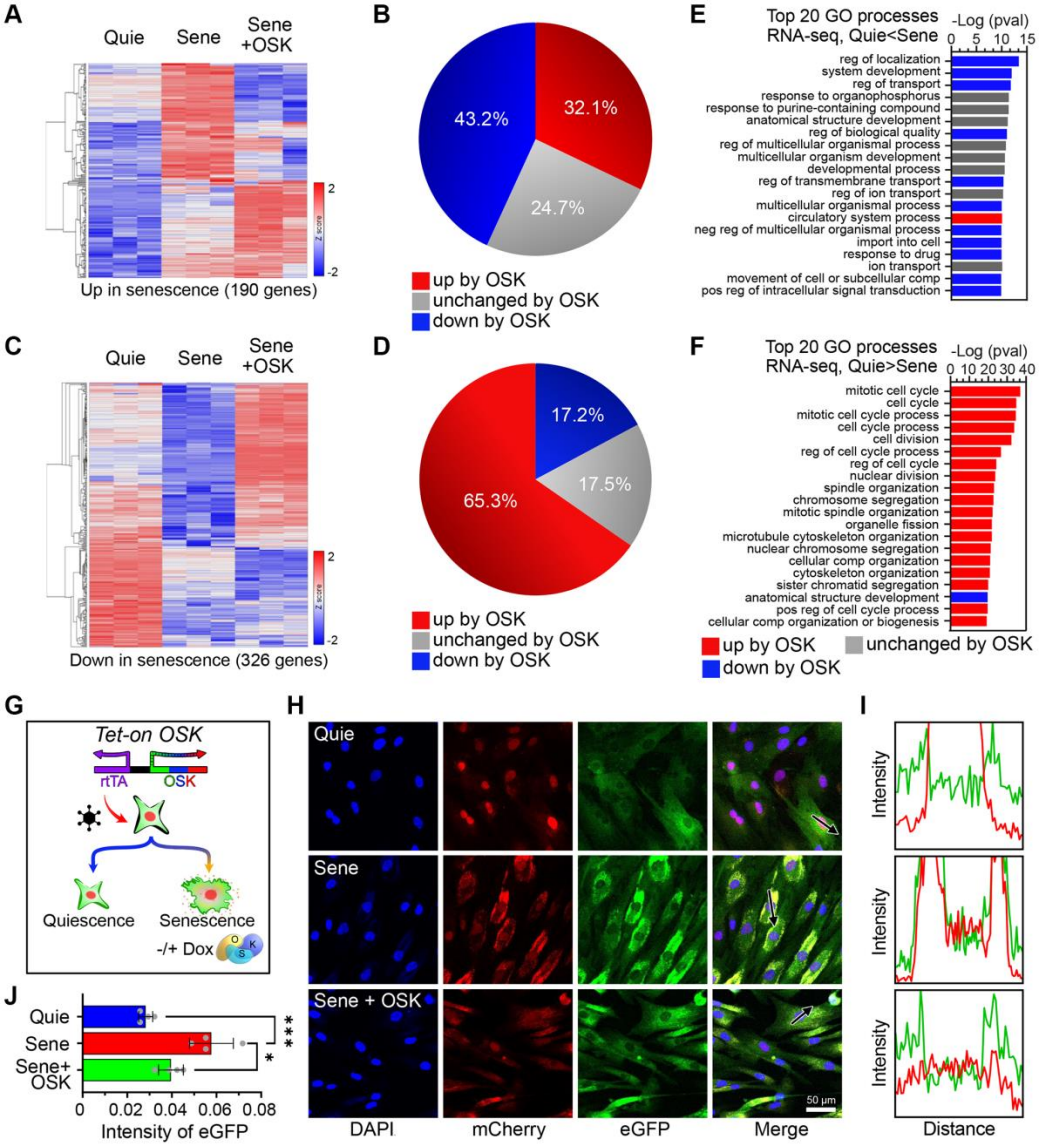
**OSK-mediated partial reprogramming ameliorates features of cellular senescence.** (**A**) Heatmaps for mRNA levels of genes upregulated by senescence (n=3, *p*-adj < 0.01, FC > 2). (**B**) Percentage of genes changed by OSK (n=3, *p*-adj < 0.05) among those upregulated by senescence. (**C**) Heatmaps for mRNA levels of genes downregulated by senescence (*p*-adj < 0.01, FC > 2). (**D**) The percentage of genes changed by OSK (*p*-adj < 0.05) among those downregulated by senescence. (**E**) Top 20 gene ontology (GO) processes of genes upregulated by senescence. The red and blue bars indicate upregulation or downregulation by OSK, respectively. (**F**) Top 20 GO processes of genes downregulated by senescence. Red and blue bars indicate upregulation or downregulation by OSK, respectively. (**G**) Schematics of the Tet-On OSK system integrated in NCC reporter system fibroblasts. (**H**) NCC signals and track of the arrows in quiescent, senescent, or senescent + OSK fibroblasts. Scale bar, 50 μm. (**I**) Fluorescence intensity profiles corresponding to the arrow in (**H**). (**J**) EGFP intensities in the cytoplasm. Data are mean ± SD. ^*^p < 0.05; ^***^p < 0.001. One-way ANOVA-Bonferroni.

Transcriptomic changes are involved in driving an aging-related decline in function and provide effective biomarkers for predicting biological and chronological age [46, 47]. To verify if these phenotypic changes reflected a more youthful epigenetic signature, we analyzed the transcriptional profile by genome-wide RNAseq. A comparison of quiescent young to quiescent old cells identified 190 genes that were significantly upregulated, and 326 genes that were significantly downregulated. Induction of OSK for four days led to reduced expression in 43.2% (82) of age-upregulated genes and increased expression in 65.3% (213) of age-downregulated genes (Figure 2A–2D and Supplementary Figure 2B). In all, nearly half of the genes changed by senescence were restored by OSK expression (Figure 2B, 2D, Supplementary Figure 2D, 2E). This finding is consistent with our previous findings and those of others, that expressing OSK in a variety of cell types and tissues, including human and mouse fibroblasts, can substantially restore the epigenetic landscape and gene expression patterns of old cells [7, 8, 26]. We call this process the EPOCH method, for epigenetic programming of old cell health.

Gene ontology (GO) analysis indicated that the top 20 GO biological processes of upregulated genes encompassed key features of aging, including dysregulation of development, localization, and transport [7], eleven of which were reversed by OSK (Figure 2E). Despite the absence of cell division in all conditions, senescence caused subtle but significant changes in cell cycle gene mRNA levels, including p21 (Supplementary Figure 2C) [49]. Numerous cell cycle- related processes were enriched with downregulated genes by senescence, and 19 of the top 20 were reversed by OSK expression (Figure 2F). The net outcome of this was the demonstration that induction of OSK partially counteracts the aging related changes resulting from senescence.

Using the NCC system, we examined the deterioration of nucleocytoplasmic integrity as cells transitioned from quiescence to senescence and the rejuvenative effects of OSK treatment on those senescent cells (Figure 2G, 2H). Cross-sectional intensity profiles of the cells were used to assess the correlation between distributions of fluorescent molecules (Figure 2I). Compared to quiescent cells, senescent cells had a significant increase in the aggregation of mCherry and eGFP, indicating disruption of nucleocytoplasmic integrity (Figure 2J). After four days of OSK treatment, NCC integrity was significantly restored in senescent cells, comparable to the quiescent, non-senescent cell population (Figure 2J). Taken together, these data show that OSK-mediated epigenetic reprogramming substantially reverses senescence-associated pathology and transcriptomic changes and that the NCC reporter system can detect rejuvenation of senescent cells by OSK.

### Reversal of senescence-associated NCC changes by reprogramming small molecules

To identify small molecules that rejuvenate old and senescent cells, we curated a list of molecules that have successfully reprogrammed human and mouse somatic cells into chemically induced pluripotent stem cells (CiPSCs) [30, 31] and tested them using the NCC assay. Again, we used fully senescent cells to avoid detecting changes due to the cell cycle or transition to senescence. Epigenetic age reversal is known to occur within a week of OSK (M)-mediated reprogramming, while the epigenetic age continuously decreases until pluripotency, reaching an approximate age of zero [50–52]. To ensure consistency, we initially tested small molecule combinations on cells within the same four-day period required for OSK to rejuvenate cells safely and consistently.

**Figure 3:**
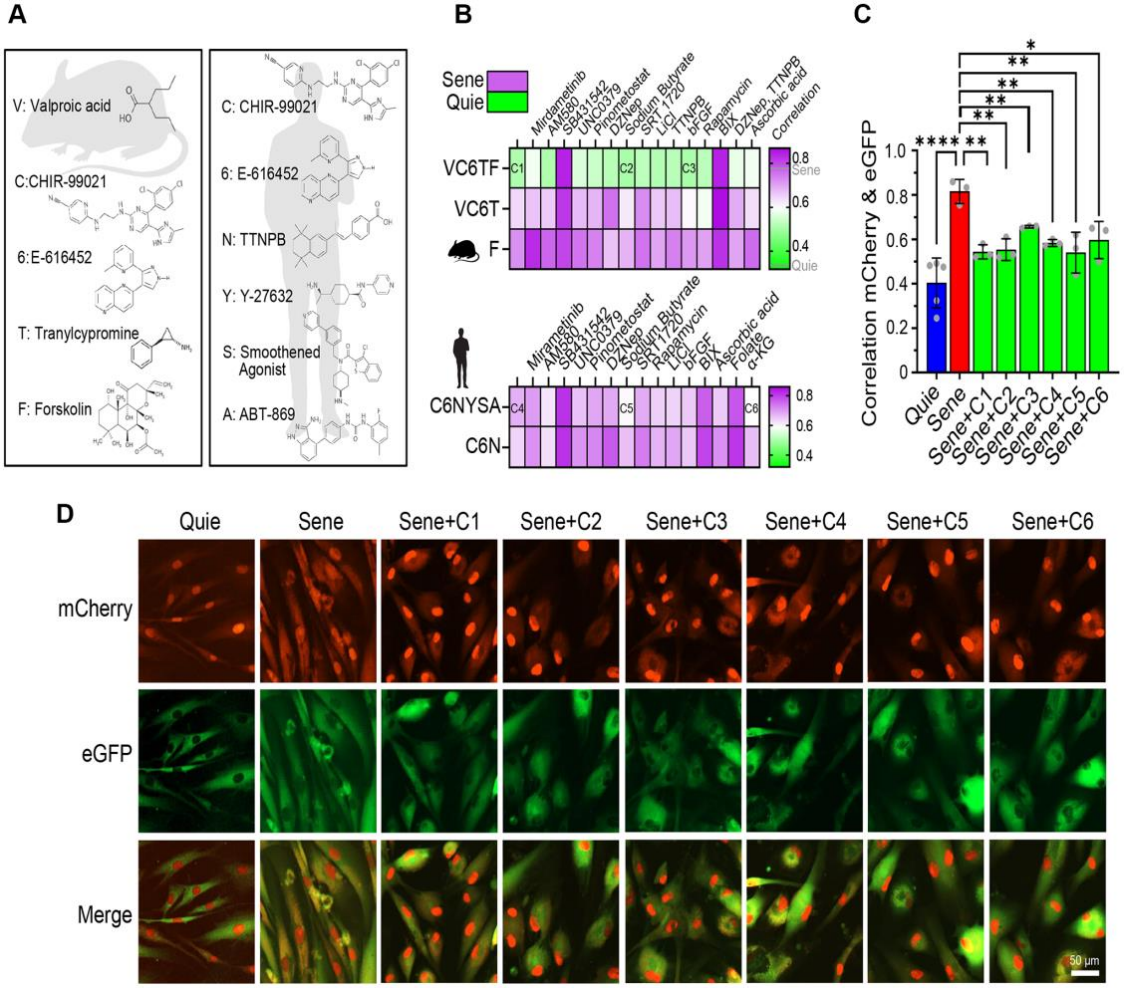
**Reprogramming small molecule cocktails restore NCC alterations in senescent cells.** (**A**) Chemical structures of small molecules of basal cocktails used to generate induced pluripotent stem cells (iPSCs) from mouse (left) or human (right) somatic cells. (**B**) Correlation heatmaps showing eGFP and mCherry colocalization in human senescent fibroblasts demonstrate the effects of 80 different combinations of small molecules (n=2). (**C**, **D**) Validation of six selected cocktails through independent experiments, showing colocalization (**C**) and representative images (**D**) of eGFP and mCherry signals. Scale bar, 50 μm. Data are mean ± SD. ^*^p < 0.05; ^**^p < 0.01; ^****^p < 0.0001. One-way ANOVA-Bonferroni.

To achieve age reduction without altering cell identity, we focused on small molecules that were likely to work in the early stages of CiPSC formation, including valproic acid (V), CHIR-99021 (C), E-616452 (6), tranylcypromine (T) and forskolin (F). Previous studies of reprogramming efficiency with small molecules demonstrated that either OCT4 alone or SKM, when combined with VC6T or F, respectively, can generate iPSCs, and VC6TF facilitates a mesenchymal-to-epithelial transition, an early stage of reprogramming in mouse cells [31, 53]. Because of the known differences in differentiation between mice and humans, we also investigated molecules that have been reported for the initiation states of generating human CiPSCs including CHIR-99021 (C), E-616452 (6), TTNPB (N), Y-27632 (Y), Smoothened Agonist (S), and ABT-869 (A) [30]. The molecules VC6TF (Cocktail 1: C1) and C6NYSA (Cocktail 4: C4) were used as basal reprogramming cocktails and supplemented with other boosters known to increase iPSC efficiency, including sodium butyrate, basic fibroblast growth factor (bFGF), and alpha ketoglutarate (α-KG) (Figure 3A, 3B, Supplementary Tables 1 and 2) [54].

Based on the fact that iPSCs can also be generated using SKM or O alone [55, 56], we evaluated the effect of the boosters on VC6T (SKM alternative) and F (O alternative). We also assessed the effect of combinations including C6N, because it has been reported that the removal of Y, S, or A from Cocktail 4 (C6NYSA) did not reduce the CiPSC efficiency [30]. Among 80 cocktails tested in the NCC assay, the VC6TF basal cocktail was the most effective at restoring the integrity of nucleocytoplasmic compartmentalization, a key sign of age-reversal (Figure 3B). A recent, unpublished study reported that 6T pre-treatment prevents senescence in human fibroblasts, and 6, T, or 6T extends the lifespan of *Caenorhabditis elegans* by up to 42.1% [57]. We, however, saw no benefit of F alone or the VC6T cocktail on reversing senescence phenotypes in our system (Figure 3B). Next, we chose six cocktails of small molecules for further investigation, three of which were based on Cocktail 1 as well as two additives (referred to as Cocktail 2 and 3) and the other three based on Cocktail 4 plus additional additives (referred to as Cocktail 5 and 6) (Supplementary Table 2). Sodium butyrate, a histone deacetylase inhibitor, was one of the most effective additives in both human and mouse cocktails (C2 and C5). Basic fibroblast growth factor (bFGF) was used for Cocktail 3, while α-KG was included in Cocktail 6. To better gauge the effect of these compounds on NCC integrity, we used Pearson’s correlation to assess the distribution of fluorescent proteins (Figure 3C, 3D). The six cocktails statistically improved compartmentalization in senescent cells, both in terms of correlation analysis (Figure 3C) and imaging of NCC signaling (Figure 3D).

For nearly two decades, the writing and maintenance of chromatin marks have been known to be critical for reprogramming [58]. For this reason, we incorporated inhibitors of established chromatin remodeling factors in our screen to investigate whether these factors represented barriers or essential drivers of rejuvenation. The rejuvenation pathway(s) initiated by C1 and C4 were both blocked by the inhibition of H3K9 methyltransferase G9a (BIX01294, 0.5 μM) and TGF-β (SB431542, 10 μM), however they were not disrupted when the H3K27 methyltransferase component of PRC2, EZH2, was inhibited (DZNep, 20 nM) (Figure 3B).

### Small molecules can reverse the age of the transcriptome with no loss of cell identity

Based on the improvement in NCC integrity, we performed RNA-seq to test the effect of these six cocktails on transcriptomic age. After treatments with the chemicals, we observed a strong overlap between genes affected by the chemical treatments and the switch from quiescence to senescence (Supplementary Figure 3A). We also observed that the two groups of cocktails generally perturbed the same populations of genes (Supplementary Figure 3A). Treatment with the chemical cocktails did not lead to fibroblasts taking on non-specific cell identity markers (Supplementary Figure 3B). Finally, we did not observe the expression of iPSC specific genes or gene modules in the RNA-seq datasets (Supplementary Figure 3C, 3D). Additionally, we performed immunofluorescence looking for signs of expression of pluripotency-related genes such as NANOG and EPCAM following all cocktail treatments but could not see any expression (Supplementary Figure 4). Collectively, these data indicate that chemical-mediated treatments are only partially reprogrammed and not fully reset to pluripotency.

**Figure 4:**
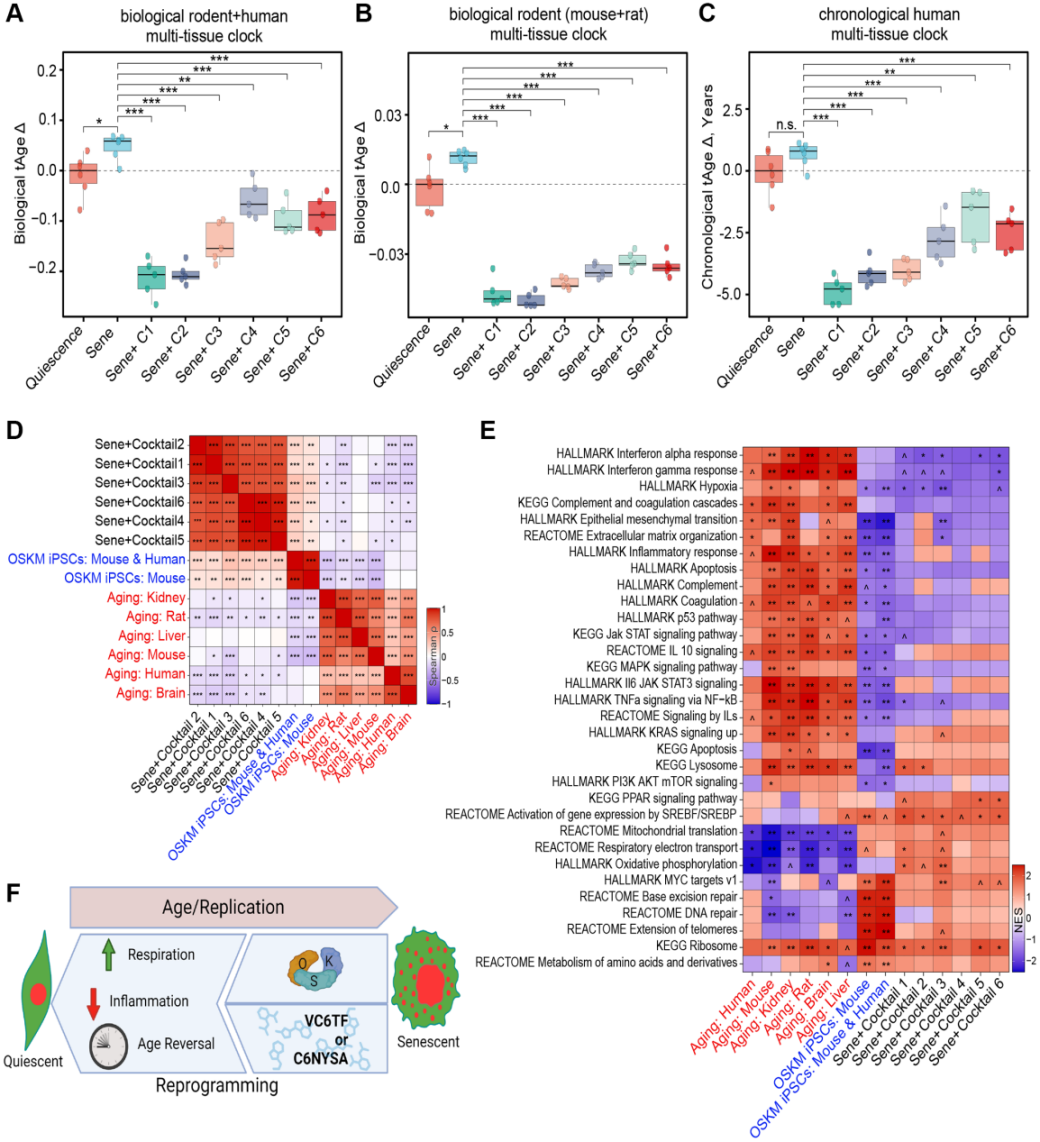
**Transcriptomic rejuvenation by reprogramming small molecule cocktails.** (**A**, **B**) Delta transcriptomic ages (tAgeΔ), as measured by a biological transcriptomic clock built on rodent and human transcriptomic data (**A**), or rodent data alone (**B**). (**C**) Delta ages, as measured by a chronological transcriptomic clock built using human data. n.s.: *p* > 0.05; ^*^p < 0.05; ^**^p < 0.01; ^***^p < 0.001. (**D**) Correlation matrix of transcriptomic differences by cocktail treatment, iPSC reprogramming, or aging. ^*^p-adj < 0.05; ^**^p-adj < 0.01; ^***^p-adj < 0.001. Benjamini-Hochberg approach. (**E**) Enrichment of pathways by cocktail treatment, iPSC reprogramming, or aging. Normalized Enrichment Score (NES) 0.05 < ^*p*-adj < 0.1; ^*^p-adj < 0.05; ^**^p-adj < 0.01; ^***^p-adj < 0.001. Benjamini-Hochberg approach. (**F**) Pictograph of study results showing that both induction of OSK and treatment with C1-6 restore NCC integrity, transcript profiles, and reversing biomarkers of health. Created using https://www.biorender.com.

We then tested the effect of these six cocktails on the transcriptomic age (tAge) of the cells using clocks trained on mouse, human, and a combined training data set 52. Relative transcriptional age was assessed using a rodent transcriptomic clock as well as a combined human and rodent transcriptomic clock (Figure 4A, 4B). The change in years of age was determined using a human-specific chronological clock (Figure 4C). Compared to quiescent cells, senescent cells had a significant increase in transcriptomic age, based on the transcriptomic clocks, consistent with previous findings assessing DNA methylation age [46, 47, 59]. Treatment of NCC cells with each of the six chemical cocktails (C1-6) resulted in statistically significant reduction of the transcriptomic age of senescent cells, with those originating from mouse studies (C1-3) generally producing a greater decrease in transcriptional age relative to the human derived cocktails (Figure 4A, 4B). The reported magnitude of the effect of all six cocktails differed between the hybrid and rodent transcriptional clocks, with the hybrid clock indicating a greater decrease in age by all six cocktails, with the rodent clock showing less variability between treatments.

All six reprogramming cocktails also significantly decreased the estimated chronological age of NCC senescent cells by several years (Figure 4C). As observed with clock-based transcriptional age estimates, C1, C2, and C3 produced the greatest effect, reducing the measured age by more than three years after only four days of treatment. For reference, the effect of this four day treatment is comparable to the total change seen after a year of a regenerative treatment described in a landmark study from 2019, which also focused on restoring epigenetic information [60].

To understand the effect of the chemical cocktails on cell identity and function, we assessed overall gene expression patterns of chemically-treated cells and compared them to old human cells [61] and OSK(M)-induced mouse and human induced pluripotent stem cells (iPSC) [52]. We expressed the correlation in gene expression between groups as a heatmap of the Spearman’s ranked correlation (Figure 4D). Despite having different chemical components, the transcriptomic profile of all six cocktails grouped most closely together, with human C4-6 and mouse C1-3-derived cocktails grouping more closely within their groups (Figure 4D). All six of the chemical treatments were positively correlated with the induced pluripotent stem cell (iPSC) populations and were negatively associated with mammalian age-related changes occurring in specific organs, such as kidney and brain, as well as across multiple tissues of mice, rats, and humans. In agreement with the transcriptomic clock analysis, mouse C1-3-derived cocktails produced a more consistent and stronger anti-aging effect on the cellular transcriptome than the human cocktails (C4-6).

Next, we performed gene set enrichment analysis (GSEA) to identify which pathways might be responsible for the similarities and differences between the chemical treatments, signatures of aging, and OSK(M)-induced iPSCs. The KEGG genes database, HALLMARK gene set collection, and Reactome pathways database were included in this analysis (Figure 4E). The anti-aging effects of chemical cocktails, especially mouse-derived ones, were associated with the upregulation of respiration associated pathways, such as oxidative phosphorylation and mitochondrial translation, as well as downregulation of hypoxia and multiple inflammation-associated pathways, such as interferon and JAK-STAT signaling, which are known to be involved in the SASP. The activation of JAK-STAT signaling by interferons and other SASP factors, for example, contributes to the complex interplay between senescent cells and their microenvironment. Together, these data show that the chemical cocktails identified in this study not only reverse the effects of senescence on NCC and make them transcriptionally younger, but they also reverse key transcriptional signatures of senescence (Figure 4F).

## DISCUSSION

In this study, we provide evidence, based on protein compartmentalization and gene expression patterns in young and senescent cells, that small molecules can reverse the transcriptomic age of cells without erasing cell identity or inducing iPSC-like states. We refer to this approach as the EPOCH method.

The effectiveness of the NCC system as an apparent surrogate biomarker for biological age reversal, with young, old, senescent, HGPS, and OSK-treated cell lines serving as controls, should set the stage for larger, more expansive screens for rejuvenation factors. Follow-up studies are underway to elucidate the cellular machinery that mediates these rejuvenative effects, with an emphasis on the mechanisms by which cells apparently write then later read a “backup copy” of earlier epigenetic information to reset chromatin structures and reestablish youthful gene expression patterns.

Disruption of NCC is a well-established effect of aging across species and is directly associated with other diseases, including amyotrophic lateral sclerosis (ALS) and frontotemporal dementia (FTD). This study shows that the expression of OSK results in a noticeable improvement in the integrity of nucleocytoplasmic compartmentalization in replicatively senescent cells. Further study into how EPOCH chemical cocktails restore NCC integrity and the partitioning of proteins may therefore offer therapeutic avenues for improving the health of older individuals and patients with age-related diseases of specific cell types and tissues. The nervous system is one example where the presence of healthy NCC is crucial for the proper functioning of tissue, and it is often affected in diseases related to aging [34–36, 62, 63]. Other methods of age control, such as the recently published Inducible Changes in the Epigenome (ICE), which has the ability to accelerate normal epigenetic aging both *in vitro* and *in vivo*, should aid in such studies [5, 7].

Transcriptomic analyses of epigenetic reprogramming by OSK and rejuvenation cocktails indicate that these interventions broadly ameliorate features of senescence, as illustrated by the striking changes in senescence-associated gene expression patterns involving inflammation, mitochondrial metabolism, lysosomal function, apoptosis, p53, and growth signaling. Furthermore, the observation from the transcriptomic clocks that all six chemical cocktails, C1-C6, decreased both biological and chronological age below that of even the non-senescent cell populations, indicates that the cocktails are potent and capable of reversing senescence-associated cellular dysfunction. Despite the differences in the composition of mouse- and human-based chemical cocktails, both affected mostly the same grouping of genes, suggesting that the effects may be operating through shared pathways.

Experiments are in progress to understand the effect of the cocktails on various cell types from young and old individuals, the results of which will inform us about the extent to which they parallel the broadly beneficial effects of OSK(M) on cells and tissues. The chemical cocktail that induced the most potent rejuvenation was VC6TF. Given that VC6TF has not been reported to be capable of fully reprogramming human cells to CiPSCs, and the maximum duration of any chemical treatment was limited to only four days, this study substantiates the notion that the rejuvenation is inherent to early phases of reprogramming and is at least partially separable from pluripotency programs [7, 8].

To fully understand how chemical epigenetic age reversal works, it will be important to identify the factors and interactions responsible and compare them to those triggered by expression of OSK. Do they work via transcription factors, OCT4, SOX2 and KLF4, or are they initiating an independent program? Additional work is also needed to determine which regulators of chromatin and transcription are involved, such as the TET enzymes, PRC1/2, and HDACs. The results from this study, and those in progress, suggest that some, but not all, of the rejuvenation mechanisms are shared between the two modes of partial reprogramming. Given that BIX01294, a G9a histone methyltransferase inhibitor, can promote full reprogramming and the formation of iPSCs, it may be that chemical rejuvenation relies on distinct pathways that establish new H3K9me1/2 marks [64]. G9a has not been extensively studied in the context of aging, except for a report citing an age-related decrease in its associated marks in certain tissues [65].

CHIR99021 is a GSK3α/β inhibitor, an effective inducer of CiPSCs and promoter of certain stem cell characteristics [66, 67]. E-616452, also known as RepSox, is a TGF-β inhibitor that has been used in experiments to replace SOX2 during epigenetic reprogramming [68, 69]. All the efficacious reprogramming chemical cocktails included these compounds, suggesting that these components together are potent contributors to the cellular rejuvenation in the treated cell populations. Various research groups have observed that chemical cocktails containing CHIR99021 and E-616452 can induce direct reprogramming between differentiated cell states [70, 71]. This is important because it suggests that the processes involved in both rewriting and replacing cellular epigenetic identity are affected by the additive effects of these chemical compounds. Moreover, independent studies have found associations with individual chemicals and reprogramming in various contexts, indicating that each component likely contributes to rejuvenation through a broad range of mechanisms [54, 72].

Valproic acid is a well-known broad-spectrum histone deacetylase inhibitor that leads to a rapid and dramatic spread of histone acetylation marks across the genome [73]. The fact that valproic acid is a critical component of many of the successful cocktails indicates that the spread of euchromatin may be an important component of partial epigenetic reprogramming [73]. Sodium butyrate is another histone deacetylase inhibitor that was effective in both human and mouse cocktails. It has been reported to improve the expression of genes associated with reprogramming, supporting the model that the regulation of histone acetylation marks is crucial for rejuvenation via reprogramming [54]. The final chemical in our most efficacious C1 cocktail, forskolin, is an activator of adenylyl cyclase that has been shown to drive reprogramming and trans differentiation, depending upon the combination of other compounds present [74, 75]. While the mechanism of action of forskolin in the context of rejuvenation remains to be identified, increasing cellular levels of cAMP and the triggering of signal cascades that are critical for adaptations in cell identity may be key.

This study focused on physiological rejuvenation and analysis of specific and well-established epigenomic signatures of aging. Whether chemical reprogramming can attenuate or reverse other hallmarks of aging and how effective it is on non-senescent cells and different cell types, tissues, and species, requires additional exploration. Experiments are in progress to determine the persistence of the rejuvenative effect after reprogramming concludes and the mechanisms by which chemical EPOCH (cEPOCH) works.

Although the potential of these and other combinations of chemicals to achieve cEPOCH is great, from treating blindness to liver failure and skin damage, in light of the toxic effects of expressing all four Yamanaka factors in mice [22], it is critical that the safety of chemical rejuvenation cocktails is tested rigorously in mammalian animal models before human trials are initiated. Although transcriptomic analysis did not indicate any developing pluripotency, based on the absence of mRNA for pro-tumorigenic genes such as NANOG and by RNA-seq analysis looking for pluripotency signatures, the only way to assess the full safety of these and other rejuvenative cocktails is to test their effects in multiple animal models, paying particular attention to signs of tissue dysplasia or cancer. To date, our experiments with genetic and chemical rejuvenation methods indicate that cells possess a barrier to becoming too young or completely losing their identity like iPSCs created using OSKM. Understanding this putative barrier would also speed the identification and development of improved age reversal methods.

The observation that genetic and chemical rejuvenation of cells is possible, restoring earlier gene expression patterns while retaining cellular identity, indicates that old cells possess information to reset their biological age, consistent with the Information Theory of Aging. Identifying how this putative information is encoded and where it resides will greatly speed the development of increasingly effective approaches to rejuvenate cells.

Future work will be directed to understanding how long the effects of these and other EPOCH treatments last *in vivo* and whether they reverse aspects of aging and extend lifespan in mice, paralleling treatment with AAV-OSK [7, 8, 26]. The assays developed in this study, combined with robotics and the increasing power of artificial intelligence, will facilitate increasingly larger screens for genes, biologics, and small molecules that safely reverse mammalian aging, and, given that aging is the single greatest contributor to human disease and suffering, these advances cannot come soon enough.

## MATERIALS AND METHODS

### Cell culture

Human fibroblasts derived from a healthy 22-year-old, 94-year-old, and a 14-year-old with HGPS were obtained from the Coriell Institute (GM23976, AG08433, and AG11498). These cells were cultured in DMEM supplemented with 20% fetal bovine serum (FBS), 1% penicillin-streptomycin (P/S), and 0.1 mM β-mercaptoethanol (β-ME). For Tet-On cells, the medium composition was adjusted to include 15% tetracycline (Tet)-free FBS instead of the usual 20% FBS. To induce replicative senescence, fibroblasts were passaged until their growth ceased completely for at least two weeks. Senescence was confirmed through various assessments, including analysis of cell morphology, cell size, and gene expression of gold-standard senescence markers.

The human iPSC (hiPSC) line AG27602 (Coriell Institute) was used as a positive control for staining of iPSC cell markers and was cultured in mTeSR™ Plus (Stem Cell Technologies, #100-1130) according to manufacturer guidelines.

### Generation of stable cells

To generate NCC-stable cells, we used FugeneHD (Promega, E2311) for transfection of pLVX-EF1alpha-2xGFP:NES-IRES-2xRFP:NLS (addgene, #71396), psPAX2 (addgene, #12260), and pMD2.G (addgene, #12259) into 293FT cells following the provided instructions. The 293FT cell medium was collected two and four days post-transfection and filtered through a 0.45-micron filter. To facilitate transduction, the collected medium was combined in a tube, concentrated using the Lenti-X™ Concentrator (Takara Bio, #631231), and added to human fibroblast medium with polybrene at a concentration of 5 μg/ml. After 24 hours, the medium was replaced with fresh medium. Following approximately one week, a subset of fibroblasts showing stable expression of mCherry and GFP were observed, and NCC-positive cells were sorted using the BD FACSAria system.

For cloning of the pLVX-UbC-rtTA-hOSK-Neo vector, the Ngn2:2A:EGFP and PuroR cassettes on pLVX-UbC-rtTA-Ngn2:2A:EGFP (addgene, #127288) were swapped with the hOCT4:2A:hSOX2:2A:hKLF4 and NeoR cassettes, respectively. NCC fibroblasts were transduced with hOSK lentivirus using the same procedure as mentioned earlier to achieve stable hOSK expression. Two days post lentiviral transduction, the cells were cultured in DMEM supplemented with 15% Tet-free FBS, 1% P/S, 0.1 mM β-ME, and 200 μg/ml G418. To induce hOSK expression, senescent fibroblasts were treated with doxycycline (2 μg/ml) for stated periods.

### Small molecule treatment

The small molecules were dissolved in suitable solvents and carefully stored according to the recommended conditions (Supplementary Table 1). To prepare for the small molecule treatment, the growth medium was changed to a low serum medium with 1% FBS, a day prior to the treatment. Fresh low serum medium was used to prepare the small molecule solution, which was thoroughly mixed before replacing the old medium in the dish. The medium containing the small molecules was completely refreshed every other day until the cells were harvested. To evaluate the alterations in NCC signals resulting from the small molecule treatment, NCC images were captured using the Cytation C10 (Agilent) imaging system. NCC correlation was calculated using Cellprofiler^®^ colocalization analysis.

### Immunofluorescence

Cells were fixed in 3.7% paraformaldehyde (PFA) for 15 minutes and washed three times with 1X PBS. Then cells were permeabilized in 0.1% Triton X-100 in PBS followed by 30 minutes of blocking with 1% bovine serum albumen (BSA) in PBS+ 0.1% Tween-20 (PBST) + 22.52 mg/mL glycine. Primary antibodies were used at the following concentrations in 1% BSA in PBST: NANOG (Invitrogen, PA5-85110) 1:200, and EPCAM (Abcam, ab71916) 1:100. Primary antibodies were incubated for 1 hour at room temperature followed by three washes of PBS. Then secondary antibodies were used at 1:1000 in 1% BSA in PBST (Goat anti-rabbit Alexa Fluor™ 647, Invitrogen A-21244 or Goat anti-rabbit Alexa Fluor™ 488, Invitrogen A-11008), incubated for one hour, and followed by three washes of PBS. Nuclear counterstaining was performed for 15 minutes using Hoechst 33342 (1:2000 in PBS) followed by a final three washes with 1X PBS. Staining was assessed by 10X wide field fluorescence imaging using the IXM-LZR and processed using Metaxpress and ImageJ.

### RNA sequencing and analysis

RNA was harvested from cells using Omega ENZA Total RNA kit and assessed for quality and integrity using an Agilent Tapestation. Library preparation and 150 bp paired-end sequencing was performed on an Illumina Novaseq by Novagene. Fastq read files were processed using FastQC. Illumina adapters were removed using TrimGalore! (Version 0.4.0, Babraham Bioinformatics), and aligned to the mm10 genome using Hisat2 (Version 2.2.1) [76]. Aligned reads were assembled using StringTie (Version 1.3.3b) [77], and expression level and transcripts were estimated. Differential expression was determined using DEseq2 [78], with FDR < 0.05.

### Signature association analysis

Association of gene expression log-fold changes induced by chemical C1-6 cocktails in human fibroblasts with established transcriptomic signatures of mammalian aging and OSK (M)-induced iPSCs was examined with Spearman correlation method as described previously [61]. The utilized signatures of aging included tissue-specific liver, kidney, and brain signatures as well as multi-tissue signatures of the mouse, rat, and human [61]. Signatures of OSKM reprogramming included genes differentially expressed during cellular reprogramming of mouse fibroblasts (mouse), and shared transcriptomic changes during OSK(M)-induced reprogramming of mouse and human fibroblasts (mouse and human) [47]. Pairwise Spearman correlations for gene expression changes induced by chemical cocktails and transcriptomic signatures of aging and OSK (M) reprogramming were calculated based on the union of top 300 genes with the lowest *p*-value for each pair of signatures.

For the identification of enriched functions affected by chemical cocktails, we performed functional GSEA [79] on a pre-ranked list of genes or proteins based on log10 (*p*-value) corrected by the sign of regulation, calculated as:


log (pv) × sgn (lfc)

where pv and lfc are *p*-value and logFC of a certain gene, respectively, obtained from edgeR output, and sgn is the signum function (equal to 1, −1 and 0 if value is positive, negative, or equal to 0, respectively). HALLMARK, KEGG, and REACTOME ontologies from the Molecular Signature Database (MSigDB) were used as gene sets for GSEA. The GSEA algorithm was performed separately for each cocktail via the fgsea package in R with 5000 permutations. *P*-values were adjusted with Benjamini-Hochberg method. An adjusted *p*-value cutoff of 0.1 was used to select statistically significant functions. A similar analysis was performed for gene expression signatures of aging and OSK (M) reprogramming.

### Transcriptomic clock analysis

To assess the transcriptomic age (tAge) of fibroblasts treated with chemical cocktails, we applied multi-tissue chronological human, lifespan-adjusted biological rodent (mouse + rat) and hybrid (mouse + rat + human) transcriptomic clocks based on the identified gene expression signatures of aging [52]. For data preprocessing, filtered RNAseq count data was passed to log transformation and scaling. The missing values corresponding to clock genes not detected in the data were imputed with the precalculated average values. Estimated sample tAges were centered around median tAge of control quiescent cells. Pairwise differences between average tAges of senescent untreated cells and either quiescent cells or senescent cells treated with C1-6 cocktails were assessed using independent *t*-tests. Resulting *p*-values were adjusted with the Bemjamini-Hochberg method.

### iPSC profiler

In order to validate that the cells treated with chemical reprogramming cocktails did not lose their fibroblast cell type identity, transcriptome of all samples were compared against that of human stem cells using SEQUIN iPSC Profiler, as previously described [80].

## Supplementary Materials

Supplementary Figures

Supplementary Tables
