# Supplementary material for: Chemically induced reprogramming to reverse cellular aging
Source: Aging (Albany NY). 2023 Jul 12;15(13):5966–89. doi: 10.18632/aging.204896 (PMC10373966; doi:10.18632/aging.204896)
Supplement: Supplementary Figures [file aging-15-204896-s001.pdf]

SUPPLEMENTARY FIGURES

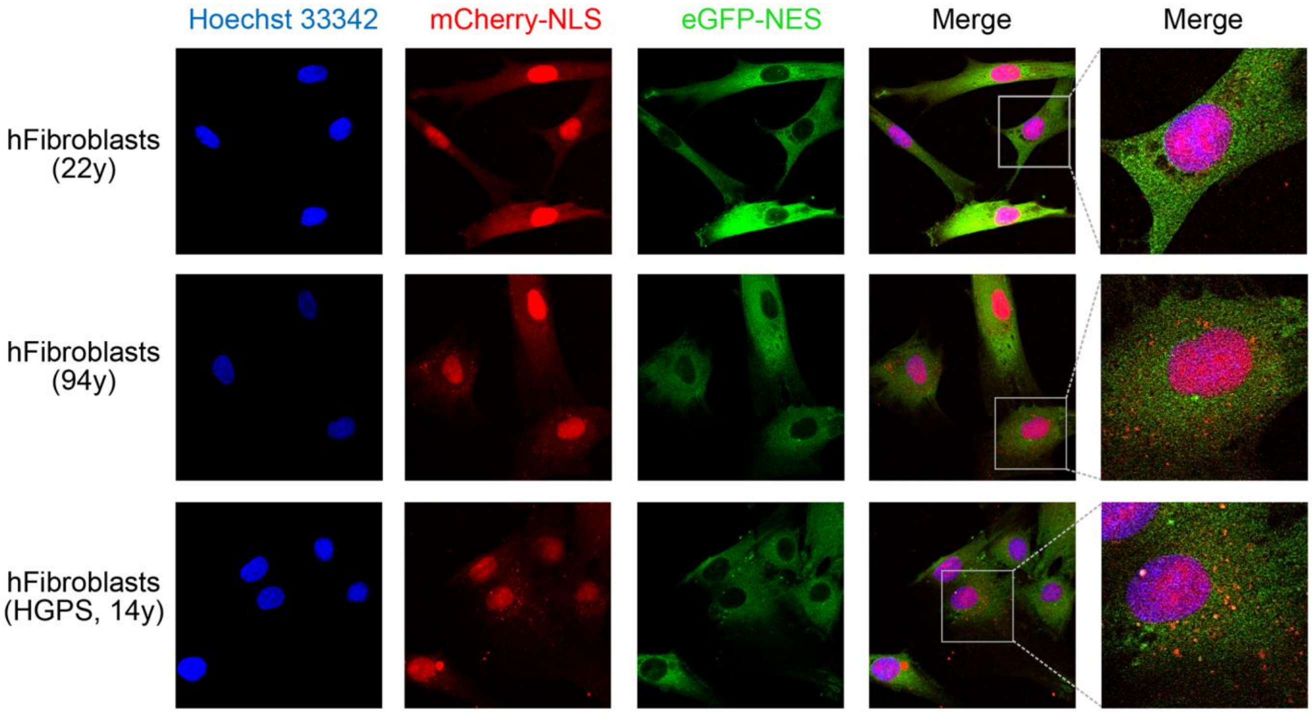

Supplementary Figure 1. NCC signals in human fibroblasts from young, old, and HGPS individuals. mCherry (red) and eGFP (green) signals in fibroblasts from 22y, 94y, or Hutchison-Guilford syndrome (HGPS) individuals with progeria. Cell nuclei were counterstained with Hoechst 33342 to define the nuclear compartment.

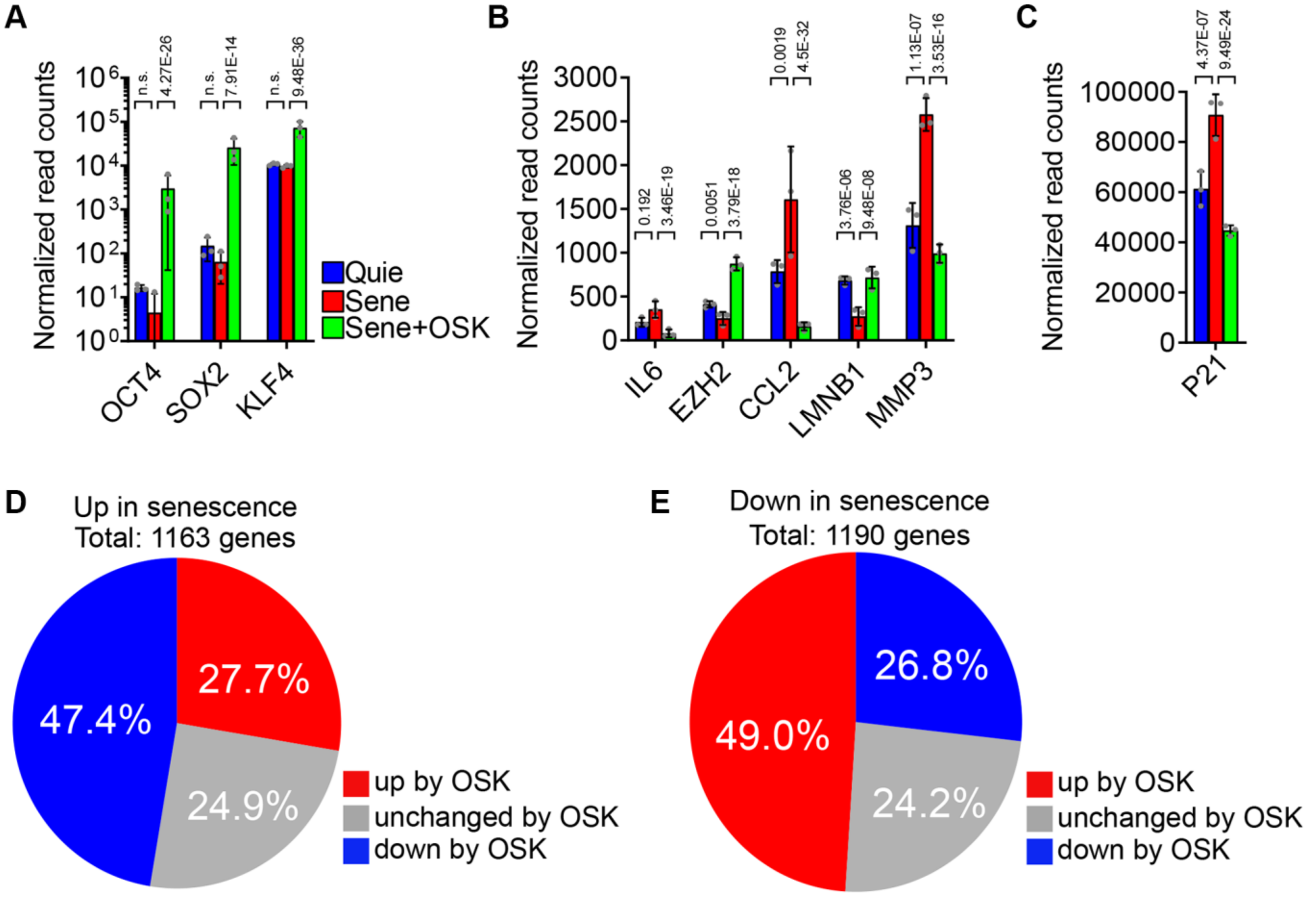

Supplementary Figure 2. mRNA levels of OSK and senescence-associated genes. (**A**–**C**) Levels of OSK in quiescent, senescent cells, and senescent cells treated with OSK. (**B**) Expression of senescence associated genes in all three conditions. (**C**) Expression of p21 (CDKN1A) in all three conditions. *P*-values have an adjusted significance threshold of $5x10^{-2}$. (**D**, **E**) The percentage of genes changed by OSK (*p*-adj < 0.05) among those upregulated and downregulated by senescence, respectively (*p*-adj < 0.05).

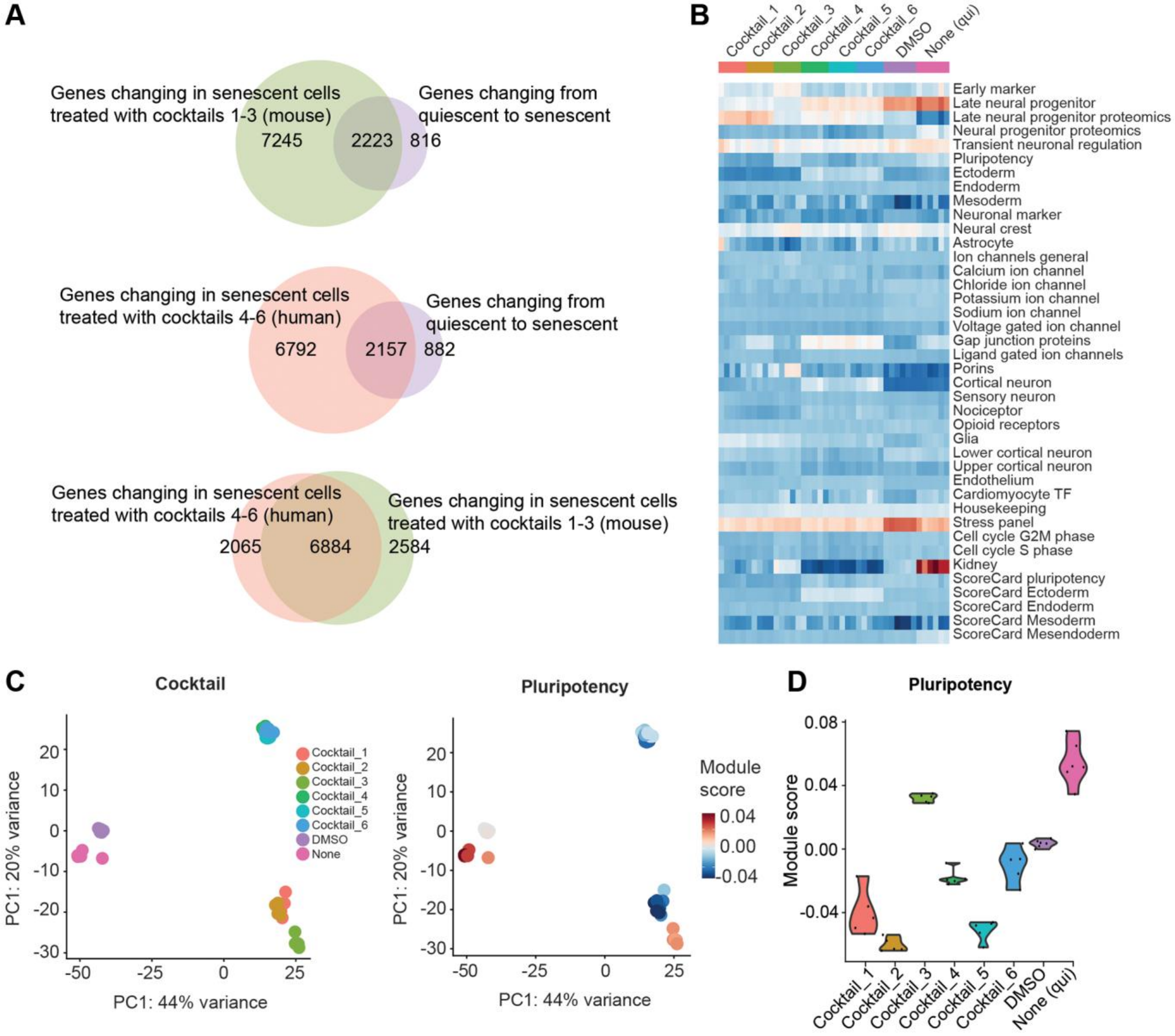

Supplementary Figure 3. Transcriptome analysis shows cocktail treatments affect mostly similar gene groups and do not promote pluripotency. (**A**) Venn diagrams of gene groups affected by mouse chemical cocktails (C1-3), human chemical cocktails (C4-6) or the transition from quiescence to senescence. (**B**) Heat-map of relative scores of each RNA-seq library condition for each of the gene expression modules as described by SEQUIN for cocktail (C1-6) comparing to DMSO-treated senescent cells and quiescent cells (None). (**C**) Principal component analysis of modules for pluripotency. (**D**) Pluripotency module scores for each RNA-seq library condition is based on iPSC Profiler (SEQUIN), comparing cocktail and DMSO control treatments in senescent cells with quiescent cells.

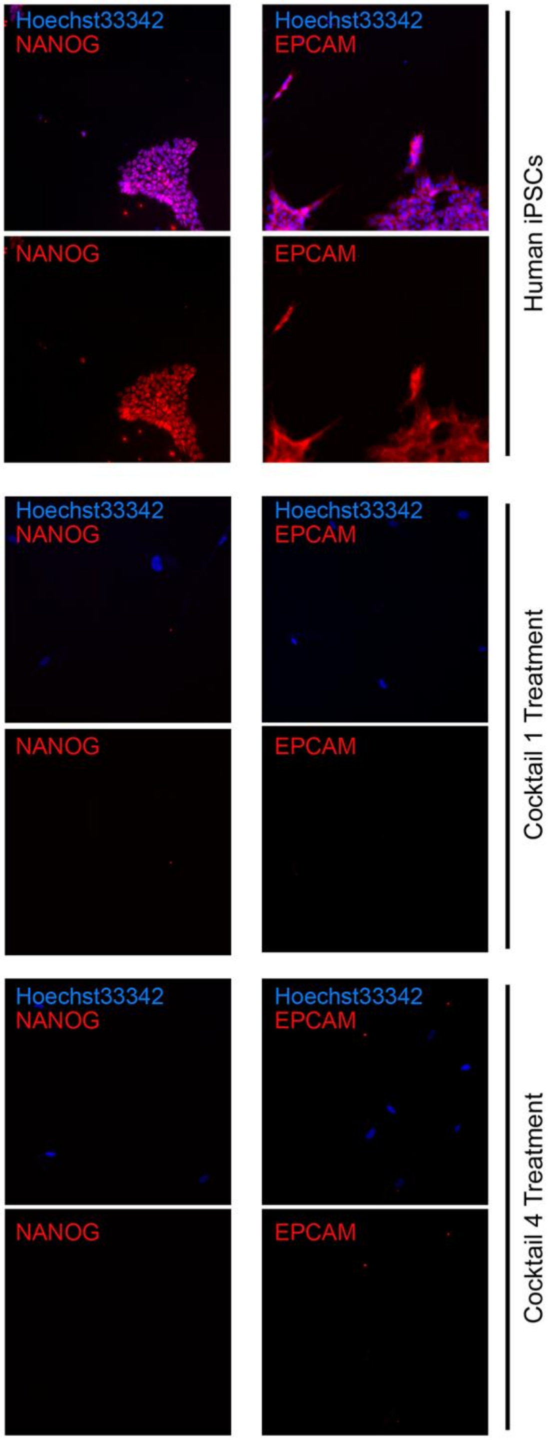

Supplementary Figure 4. Representative images of IF for pluripotency factors following chemical treatment. Representative immunofluorescence (IF) staining of either hiPSC positive control cells or senescent cells treated with cocktail 1 or 4, for markers of pluripotency (NANOG and EPCAM). IF and imaging were performed on all cells treated with the cocktails, with no observable differences between any of them for these markers.
