# Supplementary material for: Chemically induced reprogramming to reverse cellular aging
Source: Aging (Albany NY). 2023 Jul 12;15(13):5966–89. doi: 10.18632/aging.204896 (PMC10373966; doi:10.18632/aging.204896)
Supplement: Supplementary Tables [file aging-15-204896-s002.pdf]

SUPPLEMENTARY TABLES

Supplementary Table 1. Small molecule concentrations.

Name	Source	Catalog No.	Final concentration (μM)	Solvent
Valproic acid (VPA)	SIGMA	P4543-10G	250	Water
CHIR99021	SIGMA	SML1046-5MG	10	DMSO
E-616452 (RepSox)	Selleck Chemicals	S7223	10	DMSO
Tranylcypromine	ENZO	BML-EI217-0001	5	Water
Forskolin (FSK)	ENZO	BML-CN100-0010	50	DMSO
TTNPB	Selleck Chemicals	S4627	2	DMSO
Y27632	Selleck Chemicals	S1049	2	DMSO
Smoothened Agonist (SAG) HCl	Selleck Chemicals	S7779	0.5	DMSO
ABT869 (Linifanib)	Selleck Chemicals	S1003	1	DMSO
BIX	Selleck Chemicals	S8006	0.5	DMSO
Sodium Butyrate	Selleck Chemicals	S1999	200	Water
α-Ketoglutaric acid	SIGMA	K1128-25G	500	Water
L-ascorbic acid	SIGMA	95209-50G	100	Water
Folate	SIGMA	F7876-10G	0.25	Water
AM580	Selleck Chemicals	S2933	0.1	DMSO
SB431542	Selleck Chemicals	S1067	10	DMSO
Mirdametinib (PD0325901)	Selleck Chemicals	S1036	1	DMSO
LiCl	SIGMA	62476-100G	10	Water
SRT 1720	Selleck Chemicals	S1129	1	DMSO
Rapamycin	SIGMA		0.1	DMSO
Pinometostat (EPZ5676)	Selleck Chemicals	S7062	2	DMSO
UNC0379	Selleck Chemicals	S7570	1	DMSO
DZNep	MedChemExpress	HY-12186	0.02	Water
bFGF	Thermo Fisher	PHG0266	100 ng/ml	Water

Supplementary Table 2. Cocktail Combinations.

Cocktail #	Chemicals	Final concentration (μM)
1	VPA	250
	CHIR99021	10
	Repsox-616452	10
	Tranylcypromine	5
	Forskolin (FSK)	50
2	VPA	250
	CHIR99021	10
	Repsox-616452	10
	Tranylcypromine	5
	Forskolin (FSK)	50
	Sodium Butyrate	200
3	VPA	250
	CHIR99021	10
	Repsox-616452	10
	Tranylcypromine	5
	Forskolin (FSK)	50
	bFGF	100 ng/ml
4	CHIR99021	10
	Repsox-616452	10
	TTNPB	2
	Y27632	2
	SAG	0.5
	ABT869	1
5	CHIR99021	10
	Repsox-616452	10
	TTNPB	2
	Y27632	2
	SAG	0.5
	ABT869	1
	Sodium Butyrate	200
6	CHIR99021	10
	Repsox-616452	10
	TTNPB	2
	Y27632	2
	SAG	0.5
	ABT869	1
	α-KG	500
